# Repurposing Vancomycin as a Potential Antiviral Agent Against PEDV via nsp13 Helicase Inhibition

**DOI:** 10.3390/ani15070923

**Published:** 2025-03-23

**Authors:** Qiao Chen, Mengqi Yu, Jiajing Guo, Jingqi Qiu, Fei Liu, Yanke Shan

**Affiliations:** 1 Joint International Research Laboratory of Animal Health and Food Safety of Ministry of Education, Nanjing Agricultural University, Nanjing 210095, China; chenqqqqq2024@163.com (Q.C.); mengqiyu0411@163.com (M.Y.); xiaodangjia67@163.com (J.G.); qiujojo123234@163.com (J.Q.); 2 Single Molecule Biochemistry & Biomedicine Laboratory (Sinmolab), Nanjing Agricultural University, Nanjing 210095, China

**Keywords:** porcine epidemic diarrhea virus, virtual screening, vancomycin, nsp13, helicase inhibitor, antiviral drug

## Abstract

Porcine epidemic diarrhea virus (PEDV) causes a highly contagious intestinal disease with severe economic impacts on the global swine industry. The non-structural protein 13 (nsp13), a viral helicase, is essential for viral replication, making it a promising target for antiviral drug development. In this study, through virtual screening and molecular dynamics simulations, Vancomycin, a small-molecule drug also clinically used as an antibacterial agent, was identified to exhibit a stable binding affinity for PEDV nsp13. The NTPase and ATP-dependent RNA helicase activities of PEDV nsp13 were confirmed *in vitro*, and the optimal biochemical reaction conditions for its dsRNA unwinding activity were established. Further experiments demonstrated that Vancomycin effectively inhibited the dual enzymatic activities of PEDV nsp13 and reduced PEDV infections *in vitro*. This research highlights Vancomycin as a novel inhibitor of PEDV nsp13, providing valuable mechanistic insights and serving as a model for antiviral drug discovery. While this study suggests its potential for repurposing as a therapeutic agent against PEDV, further investigations are required to evaluate its feasibility *in vivo*, particularly in terms of safety, efficacy, and practical applicability.

## Simple Summary

Porcine epidemic diarrhea virus (PEDV) is an extremely virulent coronavirus in the swine industry, causing severe economic losses globally. Given the lack of effective antiviral therapies, this study identified that Vancomycin, a glycopeptide antibiotic, effectively inhibits both the NTPase and RNA helicase activities of PEDV non-structural protein 13 (nsp13) in a dose-dependent manner, resulting in highly effective reduced viral replication *in vitro*. These findings highlight the potential of Vancomycin as a candidate for antiviral drug repurposing against PEDV, offering a promising approach for controlling PEDV outbreaks.

## 1. Introduction

Porcine epidemic diarrhea virus (PEDV), an enveloped virus with a non-segmented, single-stranded, and positive-sense RNA genome, belongs to the family *Coronaviridae* and subfamily *Coronavirinae*. It is the most important swine enteric coronavirus (SECoV), causing a highly pathogenic infectious disease in piglets, characterized by enteritis and fatal watery diarrhea [1]. PEDV has become one of the most devastating viral diseases affecting swine worldwide, leading to substantial financial losses [2]. A recent serological survey conducted in the Campania region reported PEDV prevalence in wild boars, highlighting the circulation of PEDV not only in domestic pig populations but also in wild boars [3,4]. And the rapid mutation of PEDV has led to circulating strains that differ genetically from those used in vaccine production, resulting in reduced vaccine efficiency [5,6]. Consequently, these findings underscore the complexity of PEDV epidemiology and the importance of continuous monitoring to prevent future outbreaks, highlighting the urgent need for novel and effective prevention and control strategies.

While vaccines are a primary defense against viral infections, their effectiveness can be compromised due to viral mutation. In this context, antiviral drugs serve as an important therapeutic option to complement vaccines and other preventive measures and are often considered one of the final choices in managing viral infections. Research into the structures and functions of coronavirus protein has led to the identification of several antiviral targets [7]. Among these, the coronavirus non-structural protein 13 (CoV nsp13), belonging to helicase superfamily 1 (SF1), has dual enzymatic activities, including NTP hydrolysis (NTPase) activity and dsDNA/dsRNA unwinding (helicase) activity with the directionality of 5′ to 3′ [8,9]. It is through its enzymatic activities that CoV nsp13 plays a crucial role in unwinding duplex RNA for viral RNA genome synthesis [10]. Furthermore, detailed structural studies of CoV nsp13 increasingly highlight its crucial role in viral replication and transcription [11,12,13]. For instance, single-particle cryogenic-electron microscopy (cryo-EM) has revealed the structures of an nsp13_2_-RTC (replication–transcription complex with two nsp13 protomers bound) [14,15]. Notably, CoV nsp13 is highly conserved among coronaviruses, with sequence similarities of 72.4%, 67.3%, 61.3%, and 59.1% between the nsp13 of Middle East Respiratory Syndrome coronavirus (MERS-CoV) and SARS-CoV, mouse hepatitis virus (MHV), and transmissible gastroenteritis virus (TGEV), respectively [16]. Another alignment showed over 90% similarity between SARS-CoV-2 and SARS-CoV [17]. These structural biology and sequence conservation studies of the CoV nsp13 have shed light on its function of regulating viral replication and transcription, making it an attractive target for antiviral drug development.

Recent studies have identified small-molecule inhibitors targeting CoV nsp13 in the hope of developing potentially effective antiviral agents, such as SSYA10-001, which inhibits SARS-CoV and MERS-CoV replication [18,19]. During the COVID-19 pandemic, inhibitors like FPA-124, Pritelivir, and FWM-1 were reported to target SARS-CoV-2 nsp13 [20,21,22]. However, current targets of antiviral screening for PEDV are largely focused on its S protein, 3C-like protease (3CLpro), and RNA-dependent RNA polymerase (RdRp) [23,24,25], but the antiviral drug screening and characterization targeting the structure and function of PEDV nsp13 are still lacking.

In this study, we first identified Vancomycin as a potential inhibitor of PEDV nsp13 through molecular docking and molecular dynamics simulations. PEDV nsp13 was successfully expressed and purified *in vitro*, and its strong NTPase activity and ATP-dependent RNA helicase unwinding activity were characterized. We established the optimal biochemical reaction conditions for PEDV nsp13’s dsRNA unwinding activity. Further experiments demonstrated that Vancomycin effectively inhibits the dual enzymatic activities of PEDV nsp13 *in vitro* and exhibits antiviral activity against PEDV in Vero cells. Our results highlight that Vancomycin holds promise as an inhibitor of PEDV nsp13 and as an effective antiviral agent against PEDV. These findings provide valuable insights into potential therapeutic strategies for controlling PEDV infections and a mechanistic framework for antiviral drug discovery.

## 2. Materials and Methods

### 2.1. Virus and Cells

The PEDV strain CV777 (NC_003436.1), stored in our laboratory, was used in this study. Vero (African green monkey kidney) cells were cultured in Dulbecco’s Modified Eagle’s Medium (DMEM, Gibco, Grand Island, NY, USA) supplemented with 10% fetal bovine serum (FBS, Gibco, Grand Island, NY, USA) and maintained at 37 °C with 5% CO_2_. Virus culture required an additional 0.3% tryptose phosphate broth (TPB, Sigma, Livonia, MI, USA) and 3 μg/mL trypsin (Sigma, Livonia, MI, USA) in the DMEM.

### 2.2. Homology Modeling

Homology modeling was performed using the Alphafold2 module on the WeMol Cloud platform main steps included the following: (1) selecting the PEDV nsp13 amino acid sequence from the amino acid sequence of PEDV CV777 polyprotein ORF1ab (GenBank: AFQ37597.1); (2) performing sequence similarity searches in the PDB crystal structure database using NCBI BLAST (https://blast.ncbi.nlm.nih.gov/Blast.cgi, accessed on 1 February 2025) and selecting 6ZSL (resolution: 1.94 Å) as the modeling template; (3) constructing the PEDV nsp13 3D model with Alphafold2; (4) evaluating the protein-folding reliability using the SAVES6.0 Verify (eligibility criteria: over 80% 3D/1D scores are above 0.1); (5) evaluating the model residue packaging quality assessment using the STRID (eligibility criteria: whether it is consisted with secondary structure); (6) evaluating the model stereochemical parameters using PROCHECK (eligibility criteria: more than 90% of the amino acid sites are in “allowed” or “good” regions); and (7) aligning the sequence of multiple sequence of five proteins with the highest similarity with the PEDV nsp13 using NCBI BLAST Search.

### 2.3. Docking-Based Virtual Screening

Molecular docking was performed on 3038 compounds from the FDA compound library with PEDV nsp13 using Vina Autodock software (version 1.2.0, The Scripps Research Institute, La Jolla, CA, USA) with each compound docked nine times to improve accuracy. The docking results were visualized with PyMOL 2.3.2. Finally, protein-ligand interaction fingerprints (PLIF) and Proteins Plus were used to analyze the key interactions between nsp13 and the small molecules, shown in 2D and 3D images.

### 2.4. Molecular Dynamics (MD)

Molecular dynamics simulation was conducted using GROMACS 2022.3. AmberTools added the GAFF force field for small molecules, and Gaussian16 was used to hydrogenate and calculate RESP potential, with these data incorporated into the MD system topology file. The simulations were run at a static temperature of 300 K and atmospheric pressure (1 Bar) with the Amber99sb-ildn force field, using Tip3p water as the solvent and Na⁺ ions to neutralize the system charge. The system was minimized using the steepest descent method, followed by 100,000 steps of NVT and NPT equilibration. A free molecular dynamics simulation was then conducted for 100 ns with 5,000,000 steps of 2 fs each. After simulation, parameters like root mean square deviation (RMSD), radius of gyration (Rg), hydrogen bonds, and Molecular Mechanics Poisson–Boltzmann Surface Area (MM/GBSA) between Vancomycin and the nsp13 active pocket were analyzed to assess binding stability and convergence.

### 2.5. Expression and Purification of Recombinant Protein

Based on the nsp13 gene sequence in PEDV (JX188454.1) provided by GenBank, codon optimization, synthesis, and construction of pET-28a-nsp13 were performed by General Biology (Anhui, China) Co., Ltd., and *E. coli BL21 (DE3)* cells (TransGen Biotech, Beijing, China) were transformed with the plasmid. Bacterial cultures were induced with 0.5 mM IPTG when OD600 reached 0.6–0.8 and grown at 16 °C for 22 h. Cells were harvested and lysed by sonication in lysis buffer (50 mM NaH_2_PO_4_, 300 mM NaCl, pH 6.0, with 10% glycerol). Purification was performed using Ni-NTA affinity chromatography (Beyotime, Shanghai, China), and the purified nsp13 was concentrated with Amicon Ultra-15 filters (Millipore, Billerica, MA, USA) and stored at −80 °C. The concentration of purified nsp13 was measured using an enhanced BCA protein assay kit (Beyotime, Shanghai, China).

### 2.6. NTPase Assay

The nsp13 protein was incubated with 2.5 mM NTP in a reaction buffer (40 mM Tris-HCl, pH 7.5, 50 mM NaCl, 2 mM Mg^2^⁺) in a 96-well black plate with deionized water to a total volume of 20 μL. The plate was incubated at 37 °C for 30 min. Free phosphate content was quantified using the Malachite Green Phosphate Detection Kit (Beyotime, Shanghai, China). Inorganic phosphate concentrations were determined by measuring absorbance at 630 nm (A630) against known standards.

### 2.7. Preparation of RNA Duplex Substrate

The RNA duplex substrate with 5′ single-stranded overhangs was generated by annealing a 42 nt Cyanine 5 (Cy5)-labeled RNA (RNA1) with a 24 nt unlabeled RNA (RNA2) in a 1:2 ratio (21.78 kDa). The RNA1 was the Cy5-labeled strand, which would serve as the marker for the unwinding reaction. The annealing process involved mixing RNA1 and RNA2 in a 1:2 ratio in a 20 μL reaction containing 50 mM Tris, pH 8.0, and 50 mM NaCl, heating the mixture to 95 °C for 5 min, and gradually cooling it to 25 °C to form helical duplexes. To prevent the re-annealing of the unwound double-stranded RNA during helicase assays, a trap RNA (RNA3) was introduced. RNA3 has a sequence inversely complementary to RNA2, allowing it to effectively compete with RNA1 for RNA2 binding. This ensures that, once RNA2 is displaced from the duplex, it forms a stable hybrid with RNA3 instead of reannealing with RNA1. The inclusion of RNA3 improves the accuracy of helicase activity measurements by preventing the reassociation of the unwound strands. All single strands were obtained from Genscript (Shanghai, China). Table 1 lists the oligonucleotide sequences.

### 2.8. RNA Duplex Unwinding Assay

A standard RNA duplex unwinding assay was conducted by incubating 2 μM nsp13 protein with 2 nM dsRNA substrates in duplex reaction buffer containing 20 mM Tris (pH 7.5), 20 mM NaCl, 2 mM dithiothreitol, 0.1 mg/mL bovine serum albumin (BSA), 2 mM Mg^2^⁺, 2 mM ATP, and 10 nM trap RNA3 at 37 °C for 30 min. The reaction was terminated by adding an equal volume of loading buffer (0.2% SDS and 20% glycerol). The mixtures were then subjected to electrophoresis on 10% native-PAGE gels, followed by scanning with an Amersham Typhoon imaging system (Cytiva, Marlborough, MA, USA). Negative controls included non-boiled reactions (to prevent heat-induced unwinding) and ATP-free reactions (to confirm ATP dependence), while a boiled reaction mixture (95 °C) was used as the positive control, which should show full denaturation of the RNA duplex.

### 2.9. Cytotoxicity Assay

Vero cells were cultured in a 37 °C, 5% CO₂ incubator and grown to monolayers in 96-well plates. Different concentrations of Vancomycin were added to the wells in triplicate and incubated for 48 h, followed by the addition of 10 μL CCK-8 solution per well for 2 h. Absorbance was measured at 450 nm, and cell viability was calculated as follows:

Cell viability=((As−Ab)/(Ac−Ab))×100%

where As is the absorbance of Vancomycin or DMSO-treated wells, Ab is the blank control, and Ac is the untreated control.

### 2.10. Drug Inhibition of PEDV

Vero cell monolayers were treated with varying concentrations of Vancomycin (final concentrations 25, 50, and 100 μM) in DMEM with 10% FBS. DMSO-treated cells served as negative controls. After 1 h at 37 °C, cells were infected with PEDV (0.01 MOI) in FBS-free DMEM with 0.3% TPB and 3 μg/mL trypsin for 2 h. Following infection, the virus solution was discarded, and fresh medium containing Vancomycin or DMSO was added. After 12 h, samples were collected for further analysis.

### 2.11. Quantitative Real-Time PCR

Total RNA was extracted from Vero cells and reverse-transcribed into cDNA using HiScript qRT SuperMix (Vazyme, Nanjing, China). Quantitative RT-PCR was conducted with AceQ^®^ qPCR SYBR^®^ Green Master Mix (Vazyme, Nanjing, China). Primer sequences are provided in Table 2. Each reaction was performed in triplicate, with results expressed as mean ± standard deviation (SD).

### 2.12. Virus Titration

Virus titers of PEDV in Vero cells were determined according to the median tissue culture infective dose (TCID50). Vero cells were seeded into 96-well plates at a density of 10^5^ cells per well in 100 μL of medium and infected with eight-fold serial dilutions of PEDV samples (Supernatant of PEDV CV777 virus solution collected after different Vancomycin treatment in 2.10). The plates were incubated for 72 h post-inoculation at 37 °C under an atmosphere of 5% CO_2_. The viral titer was calculated in accordance with the Reed–Muench method.

### 2.13. Western Blot

Drug-treated cells were lysed in 100 μL RIPA buffer (Beyotime, Shanghai, China) with 1 mM PMSF on ice for 15 min, and lysates were collected by centrifugation. Equal amounts of protein were resolved by SDS-PAGE and transferred to a nitrocellulose membrane. The membrane was blocked with 5% non-fat milk in TBST for 2 h at room temperature; then, it was incubated with various primary antibodies: anti-PEDV N-protein (1:1000, prepared in our Laboratory), anti-GAPDH (60004-1-Ig, 1:5000; Proteintech Group, San Diego, CA, USA), and horseradish peroxidase (HRP)-conjugated goat anti-mouse secondary antibodies (1:1000; A0216, Beyotime, Shanghai, China). Finally, bound proteins were detected using the enhanced chemiluminescence (ECL) buffer (Vazyme, Nanjing, China). Proteins were detected using an enhanced chemiluminescence (ECL) buffer (Vazyme, Nanjing, China).

### 2.14. Immunofluorescence Assay

The drug-treated cells were fixed with 4% paraformaldehyde for 15 min at room temperature, washed three times with phosphate-buffered saline (PBS), permeabilized with 0.1% Triton X-100 for 5 min, and blocked with QuickBlock blocking buffer (Beyotime, Shanghai, China) for 15 min. Next, the cells were incubated with anti-PEDV N-protein primary antibody (1:200 dilutions; prepared in our Laboratory) overnight at 4 °C. After being washed three times with PBS, the cells were incubated with Alexa Fluor 488-conjugated goat anti-mouse IgG secondary antibody (1:500 dilutions, Thermo Fisher, Waltham, MA, USA) to label PEDV N-protein. Finally, Hoechst 33,342 (Thermo Fisher, Waltham, MA, USA) was used to stain cellular nuclei. After washing with PBS, the cells were visualized using an inverted fluorescence microscope.

### 2.15. Statistical Analysis

Statistical analyses were performed using GraphPad Prism (version 10.0, GraphPad Software, San Diego, CA, USA). Results are expressed as mean ± SD. Differences between groups were examined for statistical significance using one-way or two-way analysis of variance (ANOVA). The asterisks in the figures indicate significant differences (* *p* < 0.05; ** *p* < 0.01; *** *p* < 0.001; ns = not significant).

## 3. Results

### 3.1. Identifying Potential Inhibitors of PEDV nsp13 Through Virtual Screening

To identify potential inhibitors targeting PEDV nsp13, we conducted a virtual screening of 3038 FDA-approved compounds against a homology model of nsp13, which was generated and verified using Alphafold2 (Figure S1). The goal was to find compounds with a strong binding affinity to nsp13’s active site, which is crucial for viral replication. The top ten compounds with the lowest binding energies are listed in Table 3. Among these, Vancomycin was selected for further analysis due to its clinical relevance in treating gastrointestinal diseases associated with PED (Figure S2).

**Figure 1:**
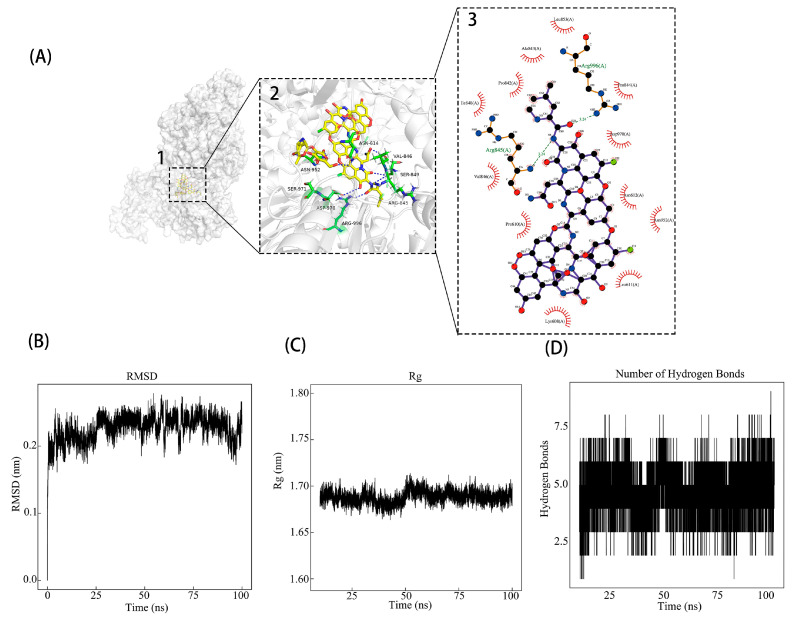
Virtual screening analysis of drugs targeting PEDV nsp13. (**A**) Binding mode of Vancomycin within the active pocket of the PEDV nsp13 protein. (1) Homology model of nsp13 and Vancomycin complex, with nsp13 represented as a gray cartoon, Vancomycin as yellow sticks, and key interacting residues as green sticks. (**2**) A 3D visualization of the Vancomycin–nsp13 binding mode. (**3**) A 2D interaction map highlighting key residues and interactions, including hydrogen bonds and hydrophobic forces. (**B**–**D**) Molecular dynamics (MD) simulation results. (**B**) Root mean square deviation (RMSD) of the backbone of nsp13 over 100 ns. (**C**) Radius of gyration (Rg) of the Vancomycin–nsp13 complex, indicating structural compactness. (**D**) Number of hydrogen bonds formed between Vancomycin and nsp13 during the simulation.

The interactions between Vancomycin and PEDV nsp13 were further analyzed using PyMOL, where key interactions such as hydrogen bonds and hydrophobic forces were visualized in both 3D and 2D formats (Figure 1A). To assess the stability of the Vancomycin–nsp13 complex, we performed a 100 ns molecular dynamics (MD) simulation. The results showed that RMSD values stabilized below 0.30 nm after 25 ns, and the change range of the whole process is far lower than the 1 nm of the general judgment standard, indicating structural stability (Figure 1B). Additionally, the radius of gyration remained between 1.65 and 1.70 nm, further confirming the stable configuration (Figure 1C). Hydrogen bond analysis showed consistent binding, with 3–5 hydrogen bonds throughout the simulation, suggesting a stable interaction between Vancomycin and nsp13 (Figure 1D). To estimate the binding affinity of Vancomycin to PEDV nsp13, we applied the MM-PBSA approach, which yielded −22.29 kcal/mol, which is far below the stronger binding affinity determination criteria for proteins and small molecules (ΔTOTAL < −7 kcal/mol) [28] (Figure S3). These results confirm Vancomycin’s strong binding affinity and stability with PEDV nsp13. Given the proximity of the binding site of Vancomycin to ATP and nucleic acid binding sites (ASN614, ASP970, and SER971), Vancomycin may interfere with both ATPase and helicase activities of PEDV nsp13, a hypothesis to be tested in subsequent experiments.

### 3.2. PEDV nsp13 Exhibits ATP Hydrolysis Activity

**Figure 2:**
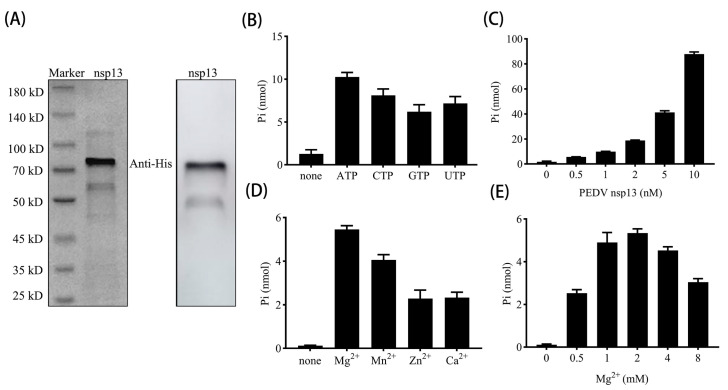
PEDV nsp13 exhibits NTPase activities. (**A**) The purified nsp13 protein was analyzed by 10% SDS-PAGE and stained with Coomassie brilliant blue (**left**) or subjected to Western blot with an anti-His antibody (**right**). (**B**) 1 nM PEDV nsp13 was incubated with various NTPs (2.5 mM each), and NTPase activity was measured as nanomoles of released inorganic phosphate (Pi) using the Malachite Green Phosphate Detection Kit. The reaction without any NTP was used as a negative control (none). (**C**) ATPase activity of PEDV nsp13 was assessed by incubating 2.5 mM ATP with increasing concentrations of nsp13. (**D**) ATPase activity was tested with 2.5 mM ATP and 2 mM of various divalent metal ions. The reaction without metal ions was used as a negative control (none). (**E**) ATP hydrolysis was measured with increasing concentrations of Mg^2+^. Error bars represent the standard deviation (SD) from three separate experiments.

To investigate whether Vancomycin is able to affect the functional activity of PEDV nsp13 as a helicase inhibitor *in vitro*, it is firstly necessary to obtain PEDV nsp13 proteins with normal biochemical activity *in vitro*. SDS-PAGE and Western blot analyses confirmed that nsp13 was successfully expressed and purified with an expected mass of 70 kDa (Figure 2A). An additional weaker band around 50 kDa was observed, which is likely a degradation product of the nsp13 protein, as this band appears in both the expressed and purified samples. However, this potential degradation product does not appear to impact the enzymatic activity of PEDV nsp13, as the full-length protein remained its expected functional activity in subsequent assays. A similar band has been observed and explained in another study on the prokaryotic expression of PEDV nsp13 [9].

NTP hydrolysis is necessary to provide energy for helicase translocation along nucleic acid substrates, and our results demonstrated that nsp13 possesses NTPase activity, hydrolyzing four kinds of NTPs. Among these, ATP was the preferred substrate and was thus selected as the primary energy source for subsequent assays (Figure 2B). Further experiments revealed that the ATPase activity increased as nsp13 concentration increased (Figure 2C). Additionally, ATPase activity was assessed in the presence of different divalent metal ions, showing that nsp13 indeed requires divalent metal ions, with efficiencies ranked as follows: Mg^2^⁺ > Mn^2^⁺ > Zn^2^⁺ ≈ Ca^2^⁺ (Figure 2D). Considering the biological relevance of Mg^2^⁺ in cellular processes, we optimized the reaction conditions and determined that 2 mM Mg^2^⁺ was sufficient for efficient ATP hydrolysis by nsp13 (Figure 2E). These findings confirm that PEDV nsp13 exhibits NTPase activity, with ATP as the preferred substrate. Furthermore, its optimal activity is dependent on specific divalent metal ions, particularly Mg^2^⁺, which serves as a co-factor to support its enzymatic function.

### 3.3. PEDV nsp13 Unwinds RNA Duplex in an ATP-Dependent Manner

**Figure 3:**
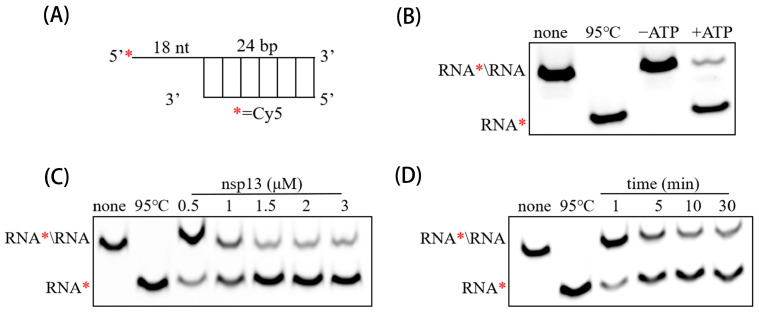
PEDV nsp13 exhibits RNA helicase activity. (**A**) Schematic of the RNA duplex substrate (RNA*/RNA). Asterisks indicate the Cy5-labeled strands. (**B**) The RNA duplex substrate (2 nM) was incubated with 2 μM nsp13, and unwinding was analyzed via gel electrophoresis and imaged on a Typhoon imager. Negative controls included non-boiled and ATP-free reaction mixtures, while a boiled reaction mixture (95 °C) served as the positive control. (**C**) The RNA duplex unwinding assay was conducted with increasing concentrations of PEDV nsp13. (**D**) Time-course analysis of RNA unwinding, with 2 μM nsp13 and 2 nM RNA substrate monitored over varying reaction times.

To confirm the RNA duplex unwinding activity of PEDV nsp13, an RNA substrate with 5′ single-stranded overhangs was constructed (Figure 3A). Results showed that the Cy5-labeled single-stranded RNA was efficiently released from the dsRNA substrate when incubated with PEDV nsp13 and ATP. In the absence of ATP, no significant unwinding was observed, confirming the ATP dependence of the reaction (Figure 3B). Additionally, increasing concentrations of PEDV nsp13 (ranging from 0.5 μM to 5 μM) enhanced the RNA duplex unwinding in a dose-dependent manner. Higher concentrations of nsp13 resulted in more Cy5-labeled RNA being released, demonstrating that the unwinding activity is directly proportional to nsp13 concentration (Figure 3C). Furthermore, the amount of released Cy5-labeled RNA increased over time, with a notable increase observed up to 30 min, after which the release rate slowed, suggesting that the unwinding reaction reaches a plateau after a certain period (Figure 3D). These results indicate that PEDV nsp13 has ATP-dependent RNA duplex unwinding activity.

### 3.4. Determining Optimal Biochemical Conditions for RNA Helicase Activity of PEDV nsp13

**Figure 4:**
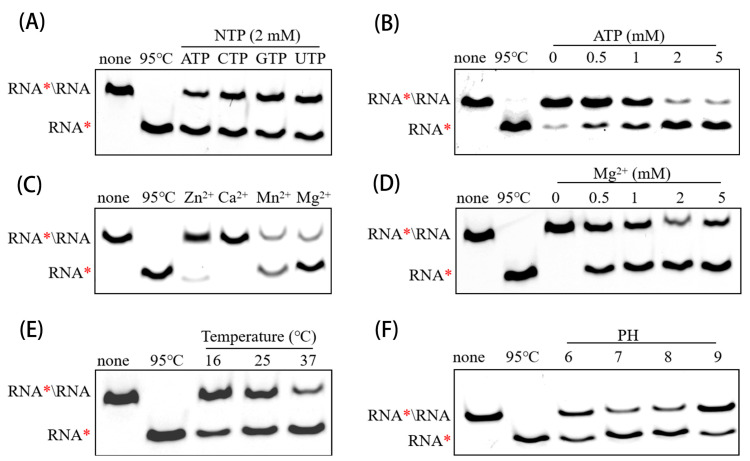
Biochemical analysis of the optimal RNA duplex unwinding activity of PEDV Nsp13. Asterisks indicate the Cy5-labeled strands. (**A**) PEDV nsp13 (2 μM) was reacted with the RNA duplex substrate (2 nM) under different biochemical conditions, including the indicated NTPs (2 mM), (**B**) increasing concentrations of ATP, (**C**) each indicated divalent metal ion (2 mM), (**D**) increasing concentrations of Mg^2+^, (**E**) varying reaction temperatures for 30 min, and (**F**) different reaction pH values. The unwinding activity was assessed as described above.

Based on the previous results, we determined the optimal reaction system for accessing the RNA duplex unwinding activity of PEDV nsp13. The enzyme was found to unwind dsRNA in the presence of four different NTPs, respectively, with ATP being the preferred energy source (Figure 4A). Increasing ATP concentrations further enhanced unwinding efficiency (Figure 4B). Similar to ATPase activity, nsp13’s helicase activity also depended on divalent metal ions, with Mg^2^⁺ or Mn^2^⁺ being essential for this activity, whereas Ca^2^⁺ and Zn^2^⁺ did not support significant helicase activity (Figure 4C). The most effective unwinding result was observed at 2 mM Mg^2^⁺ (Figure 4D). Finally, the ideal temperature and pH for unwinding were 37 °C and pH 7–8, respectively (Figure 4E,F). These results demonstrate the RNA duplex unwinding activity of PEDV nsp13 is highly sensitive to reaction conditions. The optimized biochemical system established here provides a foundation for further studies, including exploring the inhibitory effect of Vancomycin on PEDV nsp13 enzymatic activity.

### 3.5. Vancomycin Inhibits ATPase and RNA Helicase Activities of PEDV nsp13

**Figure 5:**
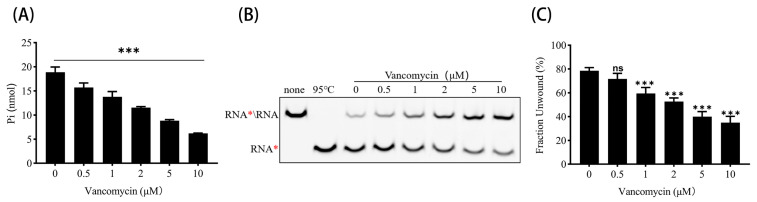
Vancomycin directly inhibited the ATPase activity and RNA duplex unwinding activity of PEDV nsp13. Asterisks indicate the Cy5-labeled strands. (**A**) PEDV nsp13 (2 μM) was incubated with 2.5 mM ATP at increasing concentrations of Vancomycin (0, 0.5, 1, 2, 5, and 10 μM), and ATPase activity was measured as described above. (**B**) PEDV nsp13 (2 μM) was reacted with an RNA duplex substrate (2 nM) under increasing concentrations of Vancomycin (0, 0.5, 1, 2, 5, and 10 μM), and the RNA duplex unwinding activity was assessed as described above. (**C**) The unwinding activity was analyzed using ImageJ (version 1.47v, National Institutes of Health, Bethesda, MD, USA) and plotted as the percentage of Cy5-labeled RNA released from the total RNA duplex (Y-axis) at the indicated concentrations of Vancomycin (X-axis). Error bars represent significant differences (*** *p* < 0.001; ns = not significant).

To further evaluate the realistic inhibitory effect of Vancomycin on PEDV nsp13, we assessed its impact on the ATPase and RNA helicase activities of the enzyme *in vitro*. As shown in Figure 5A,B, the inhibition of both activities was observed to increase with the increasing concentration of Vancomycin. Particularly, according to the statistics, the ATPase efficiency of PEDV nsp13 is significantly reduced by 0.5 μM and more Vancomycin, while the unwinding efficiency is significantly inhibited by 1 μM and more Vancomycin. Quantitative analysis revealed that Vancomycin inhibited the ATPase and helicase function by up to 50% at the highest tested concentration (Figure 5A,C). These results demonstrate that Vancomycin effectively suppresses both ATPase and RNA helicase activities of PEDV nsp13 in a concentration-dependent manner, further supporting its potential as an inhibitor of PEDV replication.

### 3.6. Vancomycin Exhibits Significant Antiviral Activity Against PEDV

**Figure 6:**
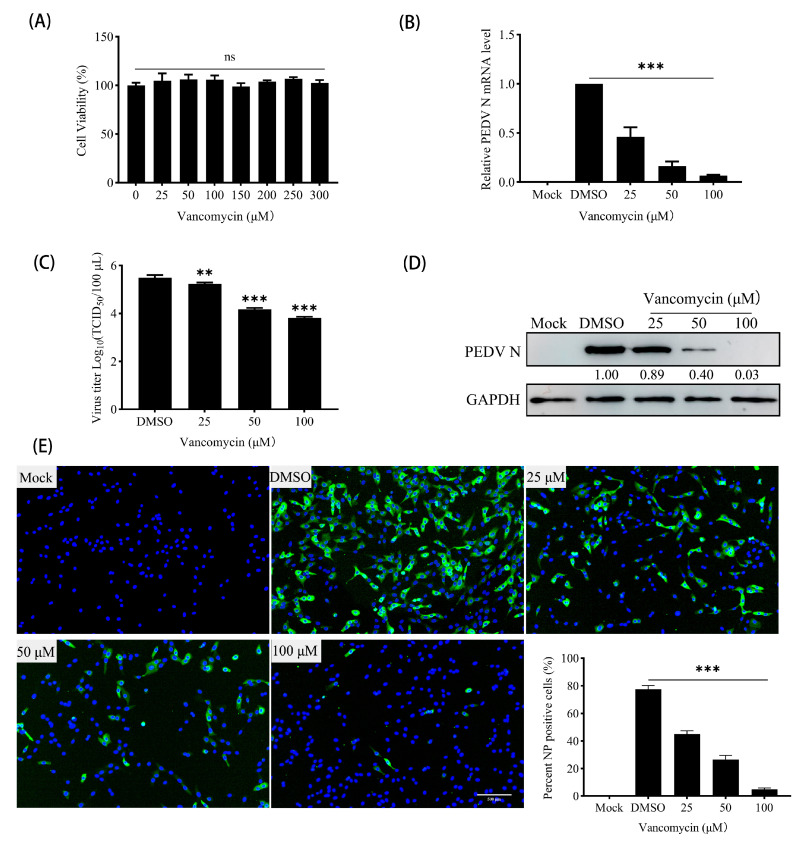
Vancomycin exhibits antiviral activity against PEDV CV777 strains in Vero cells. (**A**) Cell viability of Vero cells treated with the indicated concentrations of Vancomycin. (**B**–**E**) Vero cells were treated with the specified concentrations of Vancomycin or DMSO as a negative control and then infected with PEDV at an MOI of 0.01, and samples were collected after 12 h. (**B**) Relative levels of PEDV N mRNA were quantified by qRT-PCR, normalized to GAPDH, and expressed relative to levels in DMSO-treated cells. (**C**) PEDV (CV777 strain) viral titers were determined in Vancomycin-treated and DMSO-treated Vero cells using the TCID50 assay. (**D**) PEDV N-protein levels were evaluated by Western blot, with GAPDH as the internal control. (**E**) Levels of PEDV infection in Vero cells were assessed using immunofluorescence, with PEDV N-protein shown in green. Scale bar = 500 μm. Error bars represent SD values from three independent experiments. Asterisks in the figures indicate significant differences (** *p* < 0.01; *** *p* < 0.001; ns = not significant).

**Figure 7:**
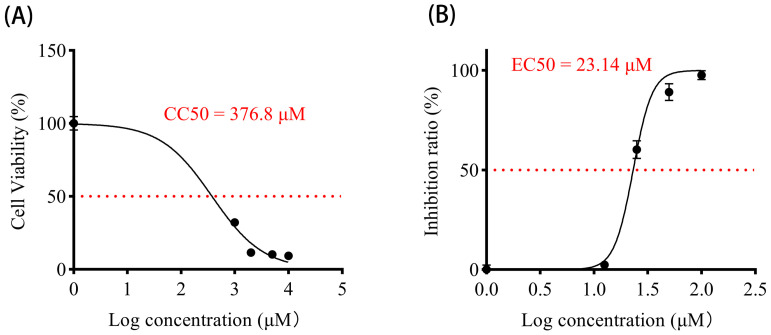
Antiviral effect of Vancomycin on PEDV. (**A**) Cell viability was measured under a series of Vancomycin concentrations using the CCK-8 assay, and the 50% Cytotoxic Concentration (CC50) values of Vancomycin were calculated based on the dose-response curve using GraphPad Prism 5. (**B**) PEDV CV777 was added at a multiplicity of infection (MOI) of 0.01 with a series of Vancomycin concentrations. Cell viability was measured, and the PEDV inhibition ratio was calculated at 24 h of Vancomycin treatment. The 50% Effective Concentration (EC50) values of Vancomycin were calculated using GraphPad Prism10 to assess inhibition ratios at different inhibitor concentrations. The error bars show the SD of the results from three replicates.

To investigate the antiviral activity of Vancomycin against PEDV, we first demonstrated that Vancomycin showed no cytotoxicity to Vero cells at working concentrations (Figure 6A), Subsequently, quantitative real-time PCR revealed that the expression of PEDV N mRNA was significantly reduced in a dose-dependent manner by Vancomycin treatment (Figure 6B). Moreover, the TCID50 assay showed that the viral titers of PEDV decreased significantly with increasing Vancomycin concentrations (Figure 6C). Similarly, Western blot analysis indicated that PEDV N-protein expression decreased with increasing concentrations of Vancomycin (Figure 6D). Additionally, immunofluorescence analysis (IFA) showed that the number of Vero cells infected with the PEDV CV777 strain gradually declined as Vancomycin concentration increased, indicating that Vancomycin significantly reduced PEDV infection in Vero cells (Figure 6E). To access the antiviral effect of Vancomycin on PEDV, the key parameters of Vancomycin such as Cytotoxic Concentration (CC50 = 376.8 μM), Effective Concentration (EC50 = 23.14 μM) and the Selective Index (SI = CC50/EC50 = 16.28) were evaluated, indicating that Vancomycin exhibits good selectivity with relatively low cytotoxicity compared to its antiviral effectiveness (Figure 7A,B). In conclusion, our data suggest that Vancomycin exhibits preliminary antiviral activity against PEDV in Vero cells.

## 4. Discussion

Coronaviruses are highly transmissible and capable of causing severe respiratory diseases with significant morbidity and mortality rates, and the zoonotic nature and ability to rapidly adapt through mutations increase the risk of future pandemics [29]. PEDV, as a typical Alphacoronavirus, poses a significant threat to the global swine industry, and although some antiviral strategies are available, there is still a lack of highly effective therapies to effectively control this devastating pathogen [6]. CoV nsp13 plays a critical role in the viral life cycle and is highly conserved across known coronaviruses, making it a common and promising target for antiviral drug development. In our study, the small-molecule compound Vancomycin was identified as a potential inhibitor of PEDV nsp13 through molecular docking assays targeting its enzymatic activity regions. Experimental results demonstrated that Vancomycin significantly suppressed the ATPase and RNA helicase activity of PEDV nsp13 *in vitro*. Moreover, subsequent analyses confirmed that Vancomycin exhibited strong antiviral effects against PEDV. This research presents a screening strategy for designing antiviral drugs that target nsp13 and establishes Vancomycin as a promising antiviral drug candidate. Additionally, these findings provide valuable insights for the development of broad-spectrum antiviral agents against coronaviruses, including PEDV.

In previous studies, it has been confirmed that CoV nsp13 has dual enzymatic activities, including NTP hydrolysis activity as well as DNA/RNA duplex unwinding activity [8,9,30]. Consequently, the main reason why most CoV nsp13 inhibitors have antiviral activity is destroying the enzymatic activities of nsp13; for example, Lu et al. identified a novel SARS-CoV-2 nsp13 inhibitor, punicalagin (PUG), which directly inhibits ATP hydrolysis to prevent nsp13 binding to DNA substrates, effectively suppressing SARS-CoV-2 replication [31]. ZINC12899676 was found to significantly inhibit PEDV replication by interfering with the NTPase activity of PEDV nsp13 *in vitro* [32]. However, screening for antiviral drugs with inhibition of both the NTPase and RNA duplex unwinding activity of PEDV nsp13 is first reported in this study, and as illustrated in Figure 1, Vancomycin was identified during the virtual screening phase as a potential inhibitor of PEDV nsp13, attributed to its strong and stable interactions with the active sites of nsp13. Molecular dynamics simulations further validated the stability of Vancomycin’s binding mode, showing that it forms hydrogen bonds and hydrophobic interactions with key residues (ASN614, ASP970, and SER971) in the functional domain of nsp13. These specific interactions likely impair the ATPase and RNA duplex unwinding activities of nsp13, which are critical during viral replication, thereby exerting antiviral effects.

PEDV nsp13 has been shown to unwind double-stranded (ds)RNA and dsDNA in a 5′ to 3′ direction and hydrolyze ATP, and the effects of metal ions, energy, and pH on its DNA duplex unwinding activity have been explored in a previous study [9]. The optimal conditions for the RNA duplex unwinding activity of PEDV nsp13 were firstly established in this study, laying the foundation for further quantitative assessment of the inhibitory effect of Vancomycin on the RNA duplex unwinding effect of PEDV nsp13 *in vitro*, thereby validating its role as a helicase inhibitor. The results demonstrated that PEDV nsp13 possesses both NTPase and ATP-dependent RNA helicase activities. Vancomycin effectively inhibited both enzymatic functions in a dose-dependent manner, highlighting its dual inhibitory capability on ATP hydrolysis and RNA duplex unwinding, differing from the previously developed PEDV nsp13 inhibitor targeting single enzyme activity. Furthermore, the reduction in PEDV infection *in vitro* following Vancomycin treatment demonstrates its function in a biological context, providing a therapeutic option for outbreak control and disease management of PEDV.

It is notable that many nsp13-targeting antiviral drugs for coronaviruses have been developed using the “drug repurposing” strategy, which involves finding new therapeutic applications for existing drugs. This approach takes advantage of pre-existing pharmacokinetic and safety data, enabling a significantly faster development process compared to traditional de novo drug discovery methods [33]. A well-known example is Zafirlukast, an FDA-approved drug for treating chronic asthma that has been proposed as an inhibitor of the nucleotide-binding site of SARS-CoV-2 nsp13 based on virtual docking, potentially blocking SARS-CoV-2 replication [34]. Diketo acids (DKAs), which have been widely investigated for their potential to inhibit cancer cell proliferation, were able to block both SARS-CoV-2 nsp13 enzymatic functions to inhibit viral replication, being active also on other human coronaviruses [35]. IOWH-032, originally developed as an ion channel inhibitor, has been found to inhibit the ATPase and helicase activities of SARS-CoV-2 nsp13 through interactions at the RNA binding interface, further lowering SARS-CoV-2 viral loads in human cells [36]. Similarly, the discovery of Vancomycin’s antiviral activity in this study expands its therapeutic scope beyond its traditional use as an antibiotic. In this study, Vancomycin’s mode of action against PEDV nsp13 does not rely on bacterial inhibition mechanisms, but rather, Vancomycin exploits its structural compatibility with PEDV nsp13 to disrupt intrinsic enzymatic activities of PEDV nsp13. Homoplastically, Wang et al. indicated that ZINC12899676 significantly inhibits the NTPase activity of PEDV by targeting its active pocket and causing its conformational change, thereby inhibiting PEDV replication in IPEC-J2 cells [32]. This unique application underscores the untapped potential of antiviral drug repositioning by structure-based virtual screening.

Historically, Vancomycin, as a glycopeptide antibiotic, has been extensively used to treat severe Gram-positive infections. However, its utility for antiviral therapy has rarely been explored [37]. Despite a large number of studies on clinical applications showing that Vancomycin has non-negligible ototoxicity and nephrotoxicity during long-term and high-dose use, it is capable of effectively reducing this side effect by combining other drugs or adjusting the dosing regimen [38]. On the other hand, Vancomycin shows lower resistance than conventional antibiotics in the course of antimicrobial therapy in swine populations [39]. Although Vancomycin has good prospects in potential antiviral applications, its direct application in pigs may be limited due to cost and potential *in vivo* toxicity concerns. So, our study primarily provides valuable mechanistic insights and serves as a model for antiviral drug discovery, rather than an immediate replacement for existing prophylactic measures, such as vaccination and surveillance.

The success of this study underscores the importance of integrating computational modeling with experimental validation in antiviral drug discovery. Virtual screening and molecular dynamics simulations were instrumental in identifying and demonstrating the power of in silico methods to streamline the drug discovery process. Such approaches can be applied to other viral targets, accelerating the identification of promising candidates for further investigation. Furthermore, this work highlights the potential of helicase inhibitors as a class of antiviral agents, and helicases are highly conserved across many RNA viruses, making them attractive targets for broad-spectrum antiviral development.

Although our study demonstrates that Vancomycin effectively inhibits PEDV nsp13 enzymatic activities and exhibits antiviral effects *in vitro*, its practical application in swine is limited. Vancomycin is considered a last-resort antibiotic in human medicine, and its use in veterinary applications is strictly regulated or banned in many countries to prevent antimicrobial resistance. Therefore, rather than proposing Vancomycin as a therapeutic agent for PEDV infection in pigs, our study primarily aims to explore the molecular mechanisms of PEDV nsp13 inhibition.

Furthermore, it would be valuable to explore the interaction between Vancomycin and PEDV nsp13 in more detail, as it is a novel helicase inhibitor. And future research could focus on improving the specificity and selectivity of Vancomycin or its derivatives, enhancing their ability to target PEDV nsp13 while minimizing toxicity and off-target effects. Additionally, optimizing the pharmacokinetic properties of Vancomycin, such as improving bioavailability and reducing potential side effects, would be crucial for expanding its therapeutic potential in veterinary settings. Moreover, exploring possible drug combinations that reduce toxicity while enhancing antiviral activity could be valuable. We should place our findings within the broader context of global efforts, particularly within the framework of the “One Health” approach promoted by the World Health Organization (WHO) for the future, which emphasizes the interconnectedness of human, animal, and environmental health, advocating for a holistic strategy to mitigate antimicrobial resistance (AMR). Further research will contribute to the broader field of antiviral drug development and may serve as a basis for designing novel nsp13 inhibitors with improved selectivity and suitability for veterinary use in the future.

## 5. Conclusions

This study identified Vancomycin as a potential inhibitor of PEDV nsp13 through virtual screening based on molecular docking and dynamics simulations. The key findings are as follows: (1) PEDV nsp13 exhibits dual enzymatic activities, including NTPase and ATP-dependent dsRNA helicase activities; (2) optimal biochemical conditions for enhanced helicase activity of PEDV nsp13 were established; (3) Vancomycin effectively inhibits both enzymatic activities of PEDV nsp13 and suppresses PEDV replication in Vero cells. These insights expand our understanding of the key viral replication enzyme nsp13 in PEDV and underscore the potential of PEDV nsp13 as a valuable target for antiviral drug development against PEDV.

## Figures and Tables

**Table 1:** Oligonucleotide sequences used in this study.

Name	5′−3′	Label	kDa
RNA1	CAUACAGUACAGGGAUCACCUCAGUUCGACUAUCGAGUAAUC	5′-Cy5	13.86
RNA2	GAUUACUCGAUAGUCGAACUGAGG		7.92
RNA3	CCUCAGUUCGACUAUCGAGUAAUC		7.92

**Table 2:** Primer sequences used in this study [26,27].

Name	5′-3′
PEDV N-Forward	TTCTTGTTTCACAGGTGGATG
PEDV N-Reverse	GCTGCTGCGTGGTTTCA
GAPDH-Forward	CCTTCCGTGTCCCTACTGCCAAC
GAPDH-Reverse	GACGCCTGCTTCACCACCTTCT

**Table 3:** The top 10 most stable compounds binding to the active site.

Compound	Binding Energy (kcal/mol)
Linaclotide	−18.1
Vancomycin	−16.3
Quinupristin	−14.8
Deslanoside	−14.0
Gamithromycin	−13.0
Thiostrepton	−12.4
Amcinonide	−11.7
BMS-927711	−11.4
Glecaprevir	−11.2
Lanreotide	−11.2

## Data Availability

The data are contained within the article.

## Supplementary Materials

The following supporting information can be downloaded at: https://www.mdpi.com/article/10.3390/ani15070923/s1, Figure S1: Model evaluation of PEDV nsp13; Figure S2: The chemical structure of Vancomycin (CAS: 1404-90-6); Figure S3: The combined free energy calculations using MM/PBSA (Molecular Mechanics Poisson-Boltzmann Surface Area) analysis of PEDV nsp13 combining with Vancomycin.
