# Supplementary material for: Repurposing Vancomycin as a Potential Antiviral Agent Against PEDV via nsp13 Helicase Inhibition
Source: Animals (Basel). 2025 Mar 23;15(7):923. doi: 10.3390/ani15070923 (PMC11987754; doi:10.3390/ani15070923)
Supplement: Supplementary file 1 [file animals-15-00923-s001.zip › animals-3489763-Supplementary.pdf]

Supplementary Materials for

Repurposing Vancomycin as a Potential Antiviral Agent Against PEDV via nsp13 Helicase Inhibition

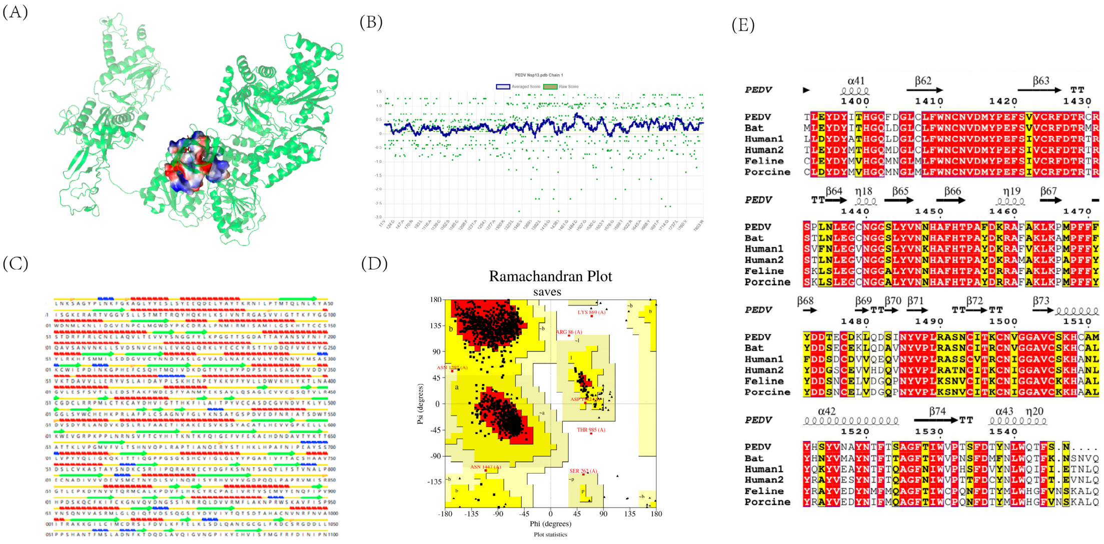

Figure S1. Model evaluation of PEDV nsp13. **(A)** Modeling structure of nsp13 protein monomer of PEDV. **(B)** Protein-folding reliability assessment using SAVES6.0 Verify Protein. **(C)** The model residue packaging quality assessment using the STRID. **(D)** The model stereochemical parameters assessment using the PROCHECK. **(E)** Multiple sequence alignment of the PEDV nsp13 amino acid sequences. Further details are provided in Materials and Methods section (2.2, 'Homology Modeling').

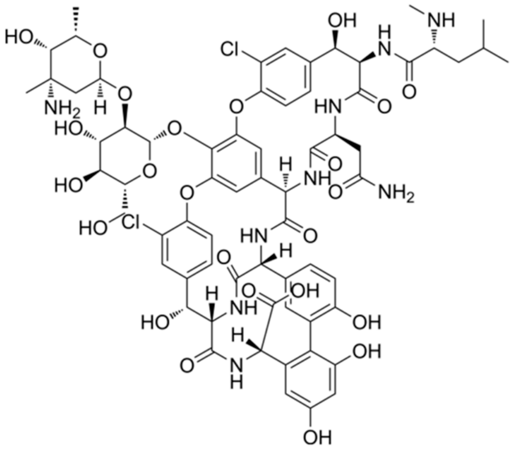

Figure S2. The chemical structure of Vancomycin (CAS: 1404-90-6).

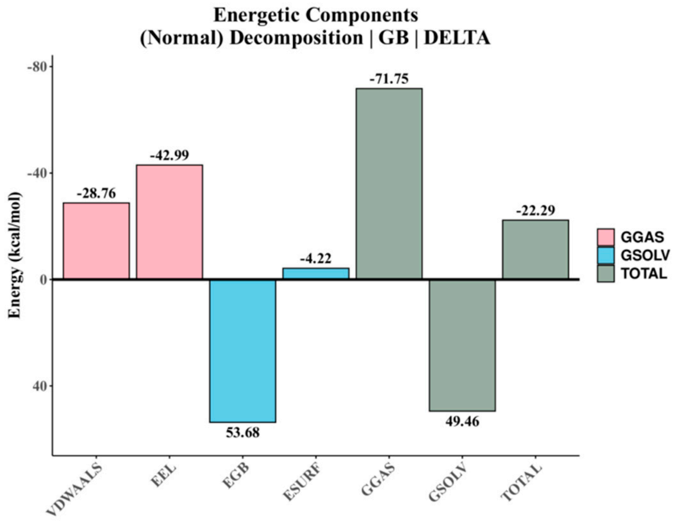

Figure S3. The combined free energy calculations using MM/PBSA (Molecular Mechanics Poisson-Boltzmann Surface Area) analysis of PEDV nsp13 combining with Vancomycin.
